# *Pseudomonas aeruginosa* strains lack tissue specificity

**DOI:** 10.1128/msystems.00076-26

**Published:** 2026-03-23

**Authors:** Alan R. Hauser

**Affiliations:** 1 Departments of Microbiology-Immunology and Medicine, Feinberg School of Medicine, Northwestern University 3270, Chicago, Illinois, USA; University of Wisconsin-Madison, Madison, Wisconsin, USA

**Keywords:** *Pseudomonas aeruginosa*, phylogenetic analysis, infectious disease

## Abstract

Bacterial pathogens vary markedly from species to species in their capacity to cause different types of infection. A recent study by Penaranda and colleagues in *mSystems* shows that the genetic backgrounds of *Pseudomonas aeruginosa* isolates do not dictate whether they infect the lungs, blood, urine, or other body sites (C. Penaranda, E. P. Brenner, A. E. Clatworthy, L. A. Cosimi, et al., mSystems 11:e01362-25, 2026, https://doi.org/10.1128/msystems.01362-25). These findings suggest that each *P. aeruginosa* strain is versatile and has the potential to infect a variety of tissues and organs.

## COMMENTARY

The ancient Greek poet Archilochus said, “a fox knows many things, but a hedgehog knows one big thing.” This statement has been interpreted to categorize entities into generalists and specialists (1), a distinction that also applies to pathogenic bacteria. A bacterium that readily infects many different organs and tissues can be thought of as a generalist, as it has the capacity to gain access to a variety of sites within the host, thwart diverse host processes aimed at expelling it, and evade local immune responses. In contrast, bacteria that have evolved to cause a specific type of infection at a particular body site may be labeled specialists. *Staphylococcus aureus* is a generalist that causes community- and hospital-acquired pneumonia, bacteremia, bone and joint infections, skin and soft tissue infections, and endocarditis. *Bordetella pertussis*, on the contrary, is a specialist that is very good at one big thing: infecting the upper respiratory tract to cause whooping cough. However, if one looks more closely, these distinctions become blurred. *Escherichia coli* infects many body sites and manifests in several different infectious presentations, but distinct pathotypes have been linked to several of these presentations. Shiga toxin-producing *E. coli* (STEC) causes hemolytic uremic syndrome; enterotoxigenic *E. coli* (ETEC) causes traveler’s diarrhea; and uropathogenic *E. coli* (UPEC) causes urinary tract infections. So, it appears that *E. coli* is a generalist comprising several specialists.

What about *Pseudomonas aeruginosa*? It acts as an opportunistic pathogen, requiring host compromise to cause disease. Under the right conditions, it readily infects the lungs, bloodstream, urinary tract, wounds, ears, and eyes. At first glance, it would, therefore, appear to be a generalist. However, *P. aeruginosa* adapts to the human body during chronic infections, such as in individuals with cystic fibrosis. Have adaptations already occurred in *P. aeruginosa* reservoirs, allowing some lineages to more readily infect certain body sites? In support of this conjecture, most *P. aeruginosa* isolates fall into one of two broad phylogenetic clades referred to as groups A and B (2–4). From a pathogenesis perspective, the bacteria in these clades are distinguished by the type III secretion system effector genes they contain. Nearly all group A strains harbor the *exoS* gene, which encodes an effector that disrupts host cell signaling and cytoskeletal structure, whereas group B strains carry the *exoU* gene, which encodes a potent cytotoxin. These two clades suggest that *P. aeruginosa* is evolving into two distinct and diverging populations of bacteria that differ in their pathogenic potential. This, in turn, suggests that *P. aeruginosa* is a specialist, with perhaps group A strains evolving to cause one type of infection and group B another.

Recently in *mSystems*, Penaranda and colleagues address the question of whether *P. aeruginosa* is a generalist or a specialist (5). Good investigators gather forensic evidence from the crime scene, and these authors are no exception. They collected 97 isolates originally cultured from different sites of infection, including the lungs, blood, urine, wounds, ears, eyes, and abscesses. They supplemented this collection with 28 environmental isolates. They next looked at the DNA evidence. They applied the granular lens of whole-genome sequencing to each isolate to create a phylogenetic tree. The authors did not find associations between the core genome phylogeny or accessory gene content of the isolates and their source (body site), suggesting that *P. aeruginosa* is indeed a generalist. These findings confirm those of earlier studies that applied PCR- and microarray-typing methodologies to smaller isolate collections (6, 7). They did, however, find that group A isolates were over-represented in the environment, consistent with a prior report that lakes, streams, and moist soil are the natural reservoirs for group A strains (8). The reservoir of group B strains, however, remains unknown and is an important unanswered question in the field. Also consistent with prior reports (9, 10), they found that environmental isolates lacked the many antibiotic resistance alleles carried by clinical isolates, suggesting that either a second reservoir exists from which patients acquire antibiotic-resistant strains or that resistance evolves rapidly in patients while they are receiving antibiotics.

To address whether isolates from the same site had similar pathogenic properties, Penaranda and colleagues measured four different virulence features of each isolate: cytotoxicity, swimming motility, biofilm formation, and pyocyanin production. They found several interesting associations. Group B isolates were more cytotoxic than group A isolates, consistent with the presence of the *exoU* gene in group B isolates (11, 12). Eye strains were more cytotoxic than environmental isolates, perhaps due to the relative paucity of group B isolates in the environment. Pulmonary strains were less motile than environmental isolates, suggesting that flagellar swimming may not be necessary for lung infections or that flagellin, a TLR5 agonist, is rapidly selected against in the lungs. Support for this notion comes from studies of *P. aeruginosa* isolates cultured from the airways of individuals with cystic fibrosis (13).

A final aspect of the study illustrates the power of whole-genome sequencing to provide a wealth of information beyond phylogeny. As mentioned, high levels of cytotoxicity were associated with group B strains, which typically contain *exoU*. The authors used their sequencing results to carefully examine six group B strains that were exceptions and not highly cytotoxic. They found that one strain lacked the *exoU* gene; one contained a mutated *exoU* gene; two had mutations in *mucA* and *exsA*, known regulators of the type III secretion system; and two had no obvious explanations. This example shows the potential of a “precision medicine,” or more specifically, a “precision clinical microbiology” approach to understanding the virulence of *P. aeruginosa* isolates.

The study by Penaranda and colleagues supports the notion that the relatively large genome (5.2–7.1 Mb) of *P. aeruginosa* provides an impressive degree of metabolic and physiological versatility to this bacterium and allows any individual lineage to inhabit a variety of niches within the human body. However, the jury is still out, and, as stated by the authors, larger studies using thousands of isolates will be necessary to definitively answer this question.
